# An economic evaluation of vector control in the age of a dengue vaccine

**DOI:** 10.1371/journal.pntd.0005785

**Published:** 2017-08-14

**Authors:** Christopher Fitzpatrick, Alexander Haines, Mathieu Bangert, Andrew Farlow, Janet Hemingway, Raman Velayudhan

**Affiliations:** 1 Department of Control of Neglected Tropical Diseases, World Health Organization, Geneva, Switzerland; 2 National Guideline Centre, Royal College of Physicians, London, United Kingdom; 3 Oxford Martin School, University of Oxford, Oxford, United Kingdom; 4 Department of Vector Biology, Liverpool School of Tropical Medicine, Liverpool, United Kingdom; University of Heidelberg, GERMANY

## Abstract

**Introduction:**

Dengue is a rapidly emerging vector-borne Neglected Tropical Disease, with a 30-fold increase in the number of cases reported since 1960. The economic cost of the illness is measured in the billions of dollars annually. Environmental change and unplanned urbanization are conspiring to raise the health and economic cost even further beyond the reach of health systems and households. The health-sector response has depended in large part on control of the *Aedes aegypti* and *Ae*. *albopictus* (mosquito) vectors. The cost-effectiveness of the first-ever dengue vaccine remains to be evaluated in the field. In this paper, we examine how it might affect the cost-effectiveness of sustained vector control.

**Methods:**

We employ a dynamic Markov model of the effects of vector control on dengue in both vectors and humans over a 15-year period, in six countries: Brazil, Columbia, Malaysia, Mexico, the Philippines, and Thailand. We evaluate the cost (direct medical costs and control programme costs) and cost-effectiveness of sustained vector control, outbreak response and/or medical case management, in the presence of a (hypothetical) highly targeted and low cost immunization strategy using a (non-hypothetical) medium-efficacy vaccine.

**Results:**

Sustained vector control using existing technologies would cost little more than outbreak response, given the associated costs of medical case management. If sustained use of existing or upcoming technologies (of similar price) reduce vector populations by 70–90%, the cost per disability-adjusted life year averted is 2013 US$ 679–1331 (best estimates) relative to no intervention. Sustained vector control could be highly cost-effective even with less effective technologies (50–70% reduction in vector populations) and in the presence of a highly targeted and low cost immunization strategy using a medium-efficacy vaccine.

**Discussion:**

Economic evaluation of the first-ever dengue vaccine is ongoing. However, even under very optimistic assumptions about a highly targeted and low cost immunization strategy, our results suggest that sustained vector control will continue to play an important role in mitigating the impact of environmental change and urbanization on human health. If additional benefits for the control of other *Aedes* borne diseases, such as Chikungunya, yellow fever and Zika fever are taken into account, the investment case is even stronger. High-burden endemic countries should proceed to map populations to be covered by sustained vector control.

## Author summary

Transmitted by the *Aedes* mosquito, dengue affects more than 100 countries and is rapidly emerging as the leading vector-borne disease. There has been a 30-fold increase in the number of cases reported since 1960. The cost of the illness to the health system and to society at large is estimated at several billions of dollars annually. The health sector response has depended in large part on controlling mosquito populations during outbreaks. Recently, the first-ever dengue vaccine received regulatory approval for use in several countries. However, its roll-out and long-term impact still needs to be evaluated in the field. In this paper, we examine how the introduction of this vaccine might alter the investment case for sustained effort to control mosquitoes. To our knowledge, this is the first economic evaluation of mosquito control in the era of the dengue vaccine. We model the cost and effects of mosquito control in Brazil, Columbia, Malaysia, Mexico, the Philippines, and Thailand. We evaluate the cost-effectiveness of mosquito control in the presence of a vaccine that does not offer full protection to all individuals. Our results suggest that sustained mosquito control will continue to be cost-effective, even if roll-out of the current vaccine is highly targeted and low-cost. These results support current global policies and strategies for the prevention and control of dengue.

## Introduction

Dengue is a rapidly emerging disease endemic in more than 100 countries, with evidence of transmission reported in 128 countries [1]. Today, hundreds of thousands of severe dengue cases arise every year, including about 20 000 deaths. The economic cost of the illness in the Americas and South-East Asia is already measured in the billions of dollars annually, including costs such as work and school days lost [2][3][4]. In the Western Pacific, between half and two-thirds of affected households have incurred debt as a result of the care they received [5][6]. Environmental change and unplanned urbanization are conspiring to raise the cost of dengue infection further beyond the reach of health systems and households. In 2014, Southern China suffered the worst outbreak of dengue fever in more than two decades; Japan saw autochthonous transmission in its first outbreak of the disease since 1945 [7,8].

In the absence of a fully effective vaccine or any treatment, dengue control has depended solely on the control of the *Aedes aegypti* and *Aedes albopictus* vectors. Current strategies include personal protection or biological, chemical, and environmental measures. In 2006, the second edition of the Disease Control Priorities Project (DCP2) put the cost per disability-adjusted life year (DALY) averted by vector control at US$ 1992–3139 (presumably in 2005 US$). Since then, the dengue economics literature suggests lower cost-effectiveness ratios ranging from 2005 US$ 227 (2013 US$ 344) per DALY averted by larval control in Cambodia to 2009 US$ 615–1267 (2013 US$ 802–1652) per DALY averted by adult mosquito control in Brazil [9][10].

A recent systematic review concluded that results were not easily comparable due to differences in methodological assumptions, and that combined control strategies remained largely unexplored [11].

The authors note that “there is growing interest in combining vector control with vaccination once a dengue vaccine becomes widely available, which recognizes that one intervention is insufficient to effectively reduce the burden of disease.” They cite results from studies with malaria and lymphatic filariasis that support the impact of simultaneously targeting vectors and pathogens.

Prior to 2015, the absence of a dengue vaccine did not preclude efforts to model the conditions under which such an immunization strategy might be cost-effective [12][13][14][15]. Prospects for a viable immunization strategy have since improved, though not without complications [16]. In December 2015, the first-ever dengue vaccine, known as chimeric yellow fever virus-dengue virus tetravalent dengue vaccine or CYD-TDV (Dengvaxia), was approved for use in the Philippines. A strategy consisting of one year of catch-up vaccinations targeting children 9–15 years of age, followed by regular vaccination of 9-year-old children, may be cost-effective at costs up to $72 from a health-care perspective and up to $78 from a societal perspective [17]. However, a review of the results of eight independent modelling groups concluded that “the potential risks of vaccination in areas with limited exposure to dengue as well as the local costs and benefits of routine vaccination are important considerations” [18].

It has been argued that, even in the era of a vaccine, the health sector response to dengue is expected to continue to depend in large part on vector surveillance and control [19,20]. In this paper, we undertake a comprehensive review and synthesis of the evidence on the cost and cost-effectiveness of dengue control interventions from the health system perspective. We appraise the cost-effectiveness of sustained vector control (including outbreak response) in the presence of a (hypothetical) highly targeted and low-cost immunization strategy using a (non-hypothetical) medium-efficacy vaccine. We consider also outbreak response and/or medical case management alone. We model a zero-cost or null scenario in which no intervention at all is implemented (not even medical case management).

We generate cost and cost-effectiveness estimates for six middle-income countries: Brazil, Columbia, Malaysia, Mexico, the Philippines, and Thailand. These countries were the focus of recent systematic reviews of epidemiological trends and make up an estimated 15% of the global burden of dengue [21]. Our approach is deliberately conservative. As the focus of the study is assessing the cost-effectiveness of vector control in the age of a dengue vaccine, a conservative approach means making generous assumptions about the efficacy and cost of the vaccine and ungenerous assumptions about the efficacy and cost of vector control.

## Methods

A dynamic compartmental model using Markov chains was built using the open source software R. The model builds on that of Luz et al. (2011) [9]. We introduce outbreaks, urbanization, and climate change and update the model within a probabilistic sensitivity analysis (PSA) framework. We generate 1000 simulations for each of 780 weekly cycles (15 years), representing the years 2016–2030, following a model burn-in period of 500 cycles. Best estimates and 95% uncertainty intervals (UIs) are obtained by the mean and the 2.5^th^ and the 97.5^th^ percentiles across all iterations. For cost-effectiveness, we report uncertainty using cost-effectiveness acceptability curves and a Net Monetary Benefit (NMB) approach.

### Baseline model with outbreaks, urbanization and climate change

The model runs two probabilistic Markov chains in parallel–one each for vector and human populations. The model is depicted in Supporting Information S1 Fig. In every cycle, the probability of a vector being infected with the dengue virus, thus moving from susceptible (S_V_) to infected (I_V_), is dependent on the number of infected humans in the previous cycle. Likewise the probability of a susceptible human (S_H_) being infected is dependent on the number of infected vectors. However, susceptible humans are separated into two categories, denoted S_H1_ and S_H2,_ representing susceptibility to first and second infections respectively, or D_H1_ and D_H2_.

New-borns enter the model in state NB_H_. New-borns have maternal immunity and therefore remain immune from dengue infection for a period of time. As maternal immunity wanes, new-borns enter the susceptible population (S_H1_). When a human recovers from their first infection (D_H1_), they enter a state of recovery from first infection (R_H1_). In state R_H1_, humans are assumed to have cross-immunity and cannot contract dengue, after which they become susceptible to a second infection (S_H2_) from a different dengue serotype. If a human contracts dengue for a second time (D_H2_)–that is, contracts a different dengue serotype–they may progress to severe dengue (SD_H_). In SD_H_, there is an elevated probability of mortality (Death). If an individual recovers from D_H2_ or SD_H_ they are assumed to no longer be susceptible (R_H2_).

The baseline model therefore allows for the effect of herd immunity (and loss thereof). As individuals move through the states, contracting dengue, the proportion of individuals that are still susceptible decreases and the population as a whole builds up herd immunity. If new individuals entering the model are less likely to contract dengue for reasons other than acquired immunity (for example, the introduction of sustained vector control, as described below), herd immunity wanes over time, at the rate of population replacement, and the probability of transmission increases.

We model symptomatic or apparent cases only. The number of cases of asymptomatic or unapparent cases is potentially much larger [22]. Several studies have demonstrated high levels of asymptomatic infection in endemic countries [23][24], leading to more severe primary symptomatic infections in those individuals [25]. Recent evidence suggest that asymptomatic viraemic individuals can infect *Aedes aegypti* [26], although there is no evidence as to whether these are then able to infect humans. We have not estimated the effect of asymptomatic infection in our models due to the limited literature available.

The (biting) female vector population is assumed to be proportional to the human population. The average number of female vectors per host is 1.0–6.0, with a sine function of period length 2π and a standard deviation of 20% [27]. In addition to seasonal variation, there is also a probability in every cycle of switching to an outbreak model of the vector population. In defining dengue outbreaks, previous studies have used moving means of the number of human cases and standard deviations of those means, but these differ between countries and even at subnational level [28].

We model outbreaks as a 100–200% increase in the vector population over a period of 2–9 weeks [29]. The increase is relative to the baseline vector population of any given intervention. This choice allows us to more directly model the effect of outbreak response. The Markov model is structured such that the increase in vector population results, through a higher probability of infective bites, in a higher number of human cases. During the burn-in period, the probability of outbreak is set such that frequency of outbreak is once every 3–5 years [30]. This range is consistent with the country reviews, although somewhat conservative with regard to Malaysia [31].

Urbanization is reflected in part by increases in the new-born and susceptible human populations, given by the crude birth rate and the urban population growth rate minus the crude birth rate, respectively. We model the combined effect of both unplanned urbanization and climate change after the burn-in period by allowing for the frequency of outbreak to drop to as low as once every 2 years (that is, frequency follows a triangular distribution with 2 and 5 as minimum and maximum, respectively, and 3 as the most likely value). Alternatively, we could have modelled climate change as an increase in the average number of female vectors per host and/or standard deviation of the sine function. However, this alternative would suggest predictable rather than unpredictable variation over time in the number of vectors and, by extension, a different type of outbreak response than we have defined below.

A list of the baseline model parameters and distributions of the probabilities underpinning the model are listed (with sources) in Table 1. Additional parameters for the intervention models are listed in Table 2, and described here. Whenever possible, we used country-specific parameter values; in practice, these were mainly available for population at risk estimates, unit cost estimates, and basic health statistics such as population, birth rates, death rates (adult and child), life expectancy, and urban growth rates. Of note, we used country-specific probabilities of death among severe dengue cases receiving care. These were based on country-reported data, and varied considerably: from a low of 0.65% in Mexico to a high of 7% in Brazil [32].

**Table 1:** Baseline model parameters, with distributions and sources.

Parameter	Distribution	Mean (with standard deviation) or range or confidence interval (CI)	Sources
**Humans**			
Population at risk in base year	Triangular	Country-specific	Most likely value is based on [33]; minimum value based on [34]; maximum value is based on [35]
Crude birth rate of population (annual)	Deterministic	Country-specific	[36]
Growth rate of urban population (annual)	Deterministic	Country-specific	[37]
Crude death rate (annual)	Deterministic	Country-specific	[38]
Probability of developing severe dengue after secondary infection (weekly)	Beta	0.048 (0.013)	[39], consistent also with [12], but we conservatively assume a zero probability after primary infection
Probability of death among severe dengue patients, without case management (weekly)	Uniform	Min = 0.05 or country-specific Max = 0.20	[40][41], with floor set at maximum of: 0.05 or country-specific probability of death among severe dengue patients, with case management
Probability of death among severe dengue patients, with case management (weekly)	Beta	Country-specific	[32]
Average age of patients who die from severe dengue	Deterministic	30	Conservative assumption, since the majority of deaths tend to occur among children, aged <15 years.[21]
Average life expectancy of general population	Deterministic	Country-specific	[42]
Rate of loss of maternal antibodies (weekly)	Gamma	0.055 (0.041)	[12]
Average duration of infectiousness, primary and secondary (weeks)	Deterministic	1	[12]; duration of illness differs (see below).
Average duration of recovery from primary infection, including cross immunity (years)	Lognormal	1.88 (95% CI 0.88–4.31)	[43], gives a weekly probability of 0.011, conservative relative to [12] which used rate of 0.0055 per day or a weekly probability of 0.038
Relative probability of being susceptible to a second infection	Triangular	Min = 0.1 Most likely = 0.66, Max = 0.75	Maximum is comparable to [12] which put the relative probability of being susceptible to i^th^ infection at (5-i)/4
Dengue disability weight	Beta	0.197 (0.004)	[44], conservative relative to [9] and [45], and same as [12]
Severe dengue disability weight	Beta	0.545 (0.004)	[44], conservative relative to [9] and [45], same as [12]; we assumed a correlation of 0.999 with non-severe dengue so that dengue disability weight does not exceed the severe dengue disability weight
Average duration of dengue illness (years)	Beta	0.019 (0.007)	[12]
Average duration of severe dengue illness (years)	Beta	0.034 (0.009)	[12]; assumed correlation of 0.999 with non-severe dengue
Discount rate for health	Uniform	0–3%	[46]
**Vectors**			
Average number of female vectors per host	Uniform	1.0–6.0	[27], with assumed sine function of period length 2π and a standard deviation of 20%
Increase in vector populations during outbreak	Uniform	100–200%	[29]
Outbreak duration (weeks), no outbreak control	Uniform	2.0–9.0	Assumption
Outbreak periodicity (years)	Triangle	Min = 2 Most likely = 3 Max = 5	Conservative assumption relative to [30]
Death rate of adult vector (weekly)	Lognormal	0.255 (0.010)	[47], which is between the values used by [12] for young and old adults
Biting rate (bites per day)	Lognormal	0.561 (0.129)	[48], conservative relative to [12], which used 0.7; conservative assumption of correlation of -0.5 with vectors per host
Probability of transmission: vector to human (per bite by an infected vector)	Beta	0.572 (0.083)	[48], conservative relative to [12], which used 0.9
Probability of transmission: human to vector (per bite of an infected human)	Beta	0.493 (0.067)	[48]
**Resource use and costs**			
Proportion of cases hospitalized, non-severe dengue (primary hospital)	Beta	0.14 (0.03)	[49]
Proportion of cases hospitalized, severe dengue (secondary hospital)	Deterministic	1.00	[50]
Duration of hospital stay, non-severe dengue (bed days)	Gamma	3.84 (0.64)	[5][51]
Ratio of duration of hospital stay, severe dengue to non-severe dengue	Deterministic	1.5	[52]
Ambulatory visits for a hospitalized case (number)	Gamma	4.42 (0.81)	[53] [51] [54]
Ambulatory visits for a non-hospitalized case (number)	Gamma	3.68 (0.76)	[51] [53] [54], with assumed correlation of 0.999 with hospitalized cases
Unit cost of hospital bed day, primary hospital (2013 US$)	Lognormal	Country-specific	See Table 3
Unit cost of hospital bed day, specialist hospital (2013 US $)	Lognormal	Country-specific	See Table 3; assumed correlation of 0.999 with primary hospital
Unit cost of ambulatory clinic visit (2013 US$)	Lognormal	Country-specific	See Table 3
Discount rate for costs	Uniform	3–6%	[46]

**Table 2:** Additional intervention model parameters, with distributions and sources.

Parameter	Distribution	Mean (with standard deviation) or range or confidence interval (CI)	Source
**Effects**			
Reduction in vector population, medium technology vector control	Uniform	50–70%	(8)(31)
Reduction in vector population, high technology vector control	Uniform	70–90%	
Outbreak length (weeks), sustained vector control and/or outbreak response	Uniform	1–2	Conservative assumption relative to [30]
Immunization coverage (annual)	Uniform	Country-specific	Minimum is 9–14 year-old population in first year of implementation (7.8–12.9% of total population in the six countries); 9 year-old population thereafter (1.3–2.2%); up to a maximum of 80% of seropositive and susceptible population
Vaccine efficacy (seropositive)	Beta	82% (95% CI 67–90%)	[55], pooled result for ≥ 9 years of age; we assume that 100% of those vaccinated in any given year are seropositive
**Costs**			
Sustained vector control (per person per month, 2013 US$)	Lognormal	Country-specific	See Table 3
Vector control during outbreak only (per person per week, 2013 US$)	Triangular	Country-specific	[53], with adjustments using proportion of labour in costs and GDP per capita to determine the minimum, maximum and most likely values
Surveillance for vector control (per person per week, 2013 US$)	Triangular	Country-specific	[56], with adjustments using proportion of labour in costs and GDP per capita to determine the minimum, maximum and most likely values
Immunization (per person immunized, 2013 US$)	Deterministic	20	Conservative assumption

### Effect of medical case management

In the presence of medical management, the fatality rate of severe dengue cases is assumed to be 0.7–7% (range of best estimates from the six countries). The fatality rate is the same regardless of whether there is vector control or immunization. In the absence of any medical case management, however, the fatality rate for severe dengue is assumed to be 5–20% [40][41].

### Effect of sustained vector control and outbreak response

Recent reviews reveal that many existing vector control technologies have not been robustly evaluated for impact on reducing human dengue cases [57,58]. By technology, we refer to any combination of strategies and intervention tools that have been costed in the economics literature; these include biological (e.g. copepods), chemical (e.g. insecticides) and environmental (e.g. screens) interventions. To our knowledge, only one study has measured the epidemiological effect of such vector control technologies. A randomized controlled trial (RCT) of community mobilization for dengue prevention demonstrated a lower risk of infection with dengue virus in children (relative risk reduction 29.5%, 95% confidence interval 3.8% to 55.3%) and fewer reports of dengue illness (24.7%, 1.8% to 51.2%) [59].

Several trials have, however, evaluated entomological impact. The above-mentioned RCT found a 51.7% (36.2% to 76.1%) reduction in the number of pupae per person (pupae found/number of residents). An earlier systematic review and meta-analysis found that the most effective method (integrated vector control) resulted in decreases of 67–88% (best estimates) in the following indices: the number of containers with larvae per 100 houses (Breteau index), the percentage of water containers positive for larvae or pupae (Container index) and the percentage of houses with water containers containing larvae or pupae (House index) [60].

The value of larval indices has been challenged, however; pupal indices may be more valuable given lower pupal mortality and higher correlation with adult densities [61]. All evidence considered, the systematic review concluded that “dengue vector control is effective in reducing vector populations, particularly when interventions use a community-based, integrated approach, which is tailored to local eco-epidemiological and sociocultural settings and combined with educational programmes to increase knowledge and understanding of best practice”.

We opted to model the effect of vector control as a reduction of the vector population (or, more generally, a reduction of the vector population capable of transmitting the virus). Entomological effects may vary significantly between settings, not least because the choice of vector control technology is country- if not community-specific [62]. We therefore considered two broad categories of vector control technology: a medium-efficacy technology that reduces vector populations by 50–70%; and a high-efficacy technology that reduces vector populations by 70–90%. We acknowledge that such reductions in adult vectors may not have been conclusively demonstrated for long-term use of existing technologies.

These above reductions are applied to periods of outbreak also, such that sustained vector control limits the increase in vector populations during the initial phases of the outbreak, before the outbreak response is deployed. We define sustained vector control as vector control activities undertaken routinely throughout the year, usually monthly, regardless of changes in the number of vectors or human cases. Outbreak response activities, in comparison, are undertaken only in response to spikes in the number of vectors or human cases and only for as long as those spikes last. During outbreaks, increases in vector populations still occur in the presence of vector control, but from a lower baseline level.

In the baseline model of outbreak, the vector population remains high for 2–9 weeks. The effect of outbreak response is modelled by a switch of the vector population to pre-outbreak levels after a lag of 1–2 weeks–the minimum time assumed to be required to detect the increase in vector populations and deploy the outbreak response. Since we are assuming that any sustained vector control programme includes also an outbreak response component, this same effect is modelled for both sustained vector control and outbreak response alone. This assumption is optimistic with regard to outbreak response and therefore conservative with regard to demonstrating the cost-effectiveness of sustained vector control relative to outbreak response.

In the absence of sustained control, vector populations return to baseline values, whereas in the presence of sustained control, vector populations return to a level determined by the vector control technology (e.g. 70–90% below baseline values, in the case of the high efficacy technology). We assume that when sustained vector control and outbreak response are introduced, it is the susceptible (rather than infected) vector population that is immediately reduced. The number of infected vectors decreases more gradually (over their 28-day life span). This assumption is pessimistic with regard to vector control and therefore conservative with regard to demonstrating the cost-effectiveness of vector control versus immunization.

### Effect of immunization

The first-ever dengue vaccine has now been licensed for use in persons aged 9–45 by countries in Asia and Latin America and is under regulatory review by others. Phase III clinical trials of CYD-TDV (Dengvaxia) have measured efficacy over 25 months from the first dose [63][64]. Pooled results (age ≥ 9) suggest efficacy of 38% (3–63%) among seronegatives and 78% (65–86%) among seropositives [16]. Unfortunately, these same trials reveal high rates of hospitalization among those who were vaccinated when seronegative. This result suggests that the immunization strategy should target the seropositive population [55].

In our model, we consider an optimistic scenario in which the immunization strategy is perfectly effective in targeting the susceptible seropositive population (further described below). The effect of immunization is modelled as a probability among individuals susceptible to a second infection (S_H2_) of moving directly to the state of recovery from a second infection (R_H2_). The same percentage reductions are applied to both outbreak and non-outbreak cycles because outbreaks are modelled as changes in vector population rather than in serotype distribution.

### Population targeted for vector control

The entire at risk human population is targeted for vector control. The broadest definition of the at-risk human population includes the urban population of the six countries. We also considered the subset living in urban slum areas. Poor urban communities typically have environmental characteristics that facilitate *Aedes spp*. breeding, including presence of refuse deposits and containers for water storage [65][66]. We also extracted from DengueMap a list of all locations from which alerts of dengue cases or deaths had been issued in 2013 [67]. We obtained the latitude and longitude coordinates for these locations using the geocode program of the R package *mapproj*. We then merged this dataset with the g-econ database [33].

We counted populations living within gecon-coded areas satisfying at least one of the following two conditions: 1) population density of more than 250 per km^2^ (non-rural settings); AND 2) average minimum temperature of no less than 5 degrees Celsius AND an average maximum temperature of no more than 36 degrees Celsius; AND 3) gross domestic product (GDP) per capita (2005 purchasing power parity) of less than US$ 10 000 (excludes areas with a level of development equivalent to a high income country); OR occurrence of a dengue alert within the 1-degree latitude by 1-degree longitude cell. These cut-offs were based on studies identified in a recent systematic review of dengue risk mapping models [68].

In PSA, the susceptible human population was represented by a triangular distribution using g-econ, urban slum and urban populations as the most likely, minimum and maximum values, respectively.

### Population targeted for immunization

Mathematical models suggest that, to achieve significant reduction in the disease burden, immunization is most effective if it includes only individuals that have been already exposed to at least one dengue virus [69]. Some countries have decided to roll out immunization by targeting specific age groups, such as all 9–14 year olds in the first year and then all (new) 9 year olds in the second year onwards. Targeting strategies may be adapted to local settings. In theory, age or other individual characteristics could be selected to either minimize the number of seronegative people or maximize the number of seropositive people vaccinated.

We assume optimistically that the susceptible seropositive population is so effectively targeted that 0% of the people that receive the vaccine are seronegative (100% are seropositive). In other words, we assume that targeting strategies are perfectly effective in avoiding vaccine-enhanced disease in vaccinated seronegative people. Furthermore, we assume that 70–80% of the population that is seropositive (and still susceptible to second infection) is contained in the population of people that are targeted over a period of 52 weeks. Of these, 80–90% are (again, optimistically) assumed compliant with vaccination (comparable to yellow fever vaccination coverage among children in Brazil).

It bears emphasizing here that the objective of this paper is to assess the cost-effectiveness of vector control in the presence of a (hypothetical) highly targeted and low-cost vaccination strategy using a (non-hypothetical) medium-efficacy vaccine; it is not to assess the cost-effectiveness of the medium efficacy vaccine itself.

### Cost of medical case management

The cost of medical management of dengue cases is based on the utilization of general health services only, or the “hotel cost” of hospital bed days and ambulatory visits excluding any laboratory tests or drugs. The hospitalization rate, duration of hospitalization, and number of ambulatory visits are provided in Table 1. We assume a hospitalization rate of 14% for non-severe dengue and 100% for severe dengue (references are provided in Table 1). Hospitalized non-severe cases are hospitalized in primary hospitals, and severe cases are hospitalized in specialist hospitals. Unit costs (best estimates and standard errors) were obtained using data and methods from WHO-CHOICE [70]. These unit cost estimates are summarized in Table 3. All symptomatic cases were assumed to receive medical management in all scenarios, regardless of whether there was sustained vector control, outbreak response and/or immunization.

**Table 3:** Country-specific unit cost estimates (best and 95% uncertainty interval), 2013 US$.

**Country**	**Ambulatory clinic visit**			**Hospital bed day, primary**			**Hospital bed day, specialist**			**Sustained vector control** **(per capita per month)**		
	Best	Low	High	Best	Low	High	Best	Low	High	Best	Low	High
BRA	7.32	1.66	23.55	52.74	20.25	117.37	63.56	25.36	138.18	0.050	0.026	0.087
COL	48.64	9.50	142.11	193.25	75.75	405.19	233.30	85.03	501.79	0.057	0.033	0.091
MEX	37.73	8.52	116.51	338.60	129.77	736.02	411.36	156.78	878.56	0.054	0.029	0.095
MYS	27.43	5.96	79.04	239.75	91.71	521.26	299.40	114.71	600.06	0.060	0.034	0.100
PHL	9.87	2.13	30.02	58.84	23.36	125.25	70.85	27.64	154.62	0.042	0.022	0.074
THA	18.29	3.53	54.42	141.55	55.27	289.73	169.24	65.69	370.75	0.055	0.033	0.088

Brazil (BRA), Colombia (COL), Mexico (MEX), Malaysia (MYS), Philippines (PHL) and Thailand (THA).

### Cost of sustained vector control and outbreak response

We conducted a search of the literature on the cost of sustained vector control interventions and identified eight studies with primary data, from 12 countries, considering different biological, chemical, and environmental measures. A subsequent systematic review revealed no additional studies [11]. We extracted data on costs as well as populations or households covered. Costs were converted to per capita terms and inflated to 2013 US$ using GDP deflators. These unit costs were then modelled in a multivariate log-log regression on population covered and GDP per capita. More than 50% of the variation in unit cost between the studies was explained by these two variables alone, driven by a strongly negative relationship with population. In addition to economies of scale, it is likely that higher cost biological and environmental measures were only implemented at smaller scale, in targeted communities. A plot of the data and regression model results are available in Supporting Information S2 Fig and Supporting Information S1 Table.

We calculated means and standard errors for the predictions for the six countries considered in this study, using their urban populations and GDP per capita. For each country, 1000 values of (log) unit cost were drawn from a normal distribution, and then exponentiated. These unit cost estimates are summarized in Table 3. The best estimates are in the range of about 2013 US$ 0.04–0.05 per person per month–similar to the cost of larviciding and/or adulticiding programs described in studies from Brazil, Cambodia, Venezuela, and Thailand. The confidence intervals are wide, however, with highs of up to 2013 US$ 0.06–0.09 allowing for smaller scale biological and environmental measures. These unit costs are similar to those described in studies from Guatemala, Kenya, Mexico, Myanmar, and the Philippines, published prior to 2013. No costing studies have been undertaken for technologies under development using Release of Insects with Dominant Lethality (RIDL) or Wolbachia symbiont infection of vectors.

The cost of outbreak response was taken from a study from Panama [53]. That study found that the cost of vector control during an outbreak, including larviciding and adulticiding, was about 2005 US$ 0.035 per person per week. The Panama costs are conservative relative to a more comprehensive costing of outbreak response in Cuba, including health education and/or replacement of defective water tanks [71][72]. Similarly, the Panama costs are conservative relative to environmental and/or chemical interventions triggered by case reports in Malaysia and Thailand [73][74]. We considered adjustments for our six countries using the proportion of labour in costs (71%) and GDP per capita relative to that of Panama in the year of the study. Minimum, most likely, and maximum values were obtained considering adjustments for GDP per capita only, or GDP per capita and the labour proportion, or no adjustment at all.

We assumed that a sustained vector control programme would implement extraordinary outbreak response interventions in the midst of an outbreak; therefore, during an outbreak, the total cost of sustained vector control includes the cost of both the sustained vector control and outbreak response interventions. To the cost of both sustained vector control and outbreak response alone we also added the cost of sustained surveillance, based on a study from Brazil, at a cost of 2013 US$ 0.014 per person per week. Again we generated country-specific minimum, most likely, and maximum values using the proportion of labour in costs (39%) and relative GDP per capita [56].

### Cost of immunization

Since the cost of a future vaccine is unknown, the cost per person vaccinated was assumed to be about 2013 US$ 20. This unit cost is assumed to include the cost of screening for seropositivity, or whatever the cost of the perfectly effective targeting strategy, as well as administration of the vaccine itself. It is purposefully optimistic. An earlier study considered a range of US$ 10–300 per unit for the cost of vaccine production alone [12]. In most countries, US$ 20 is less than the cost of the 1–3 outpatient visits that would be required for administration alone (Table 3). It is also considerably lower than the maximum of the median willingness-to-pay results (US$ 70) from Vietnam, Thailand, and Colombia [75].

## Results

### Effects

**Fig 1:**
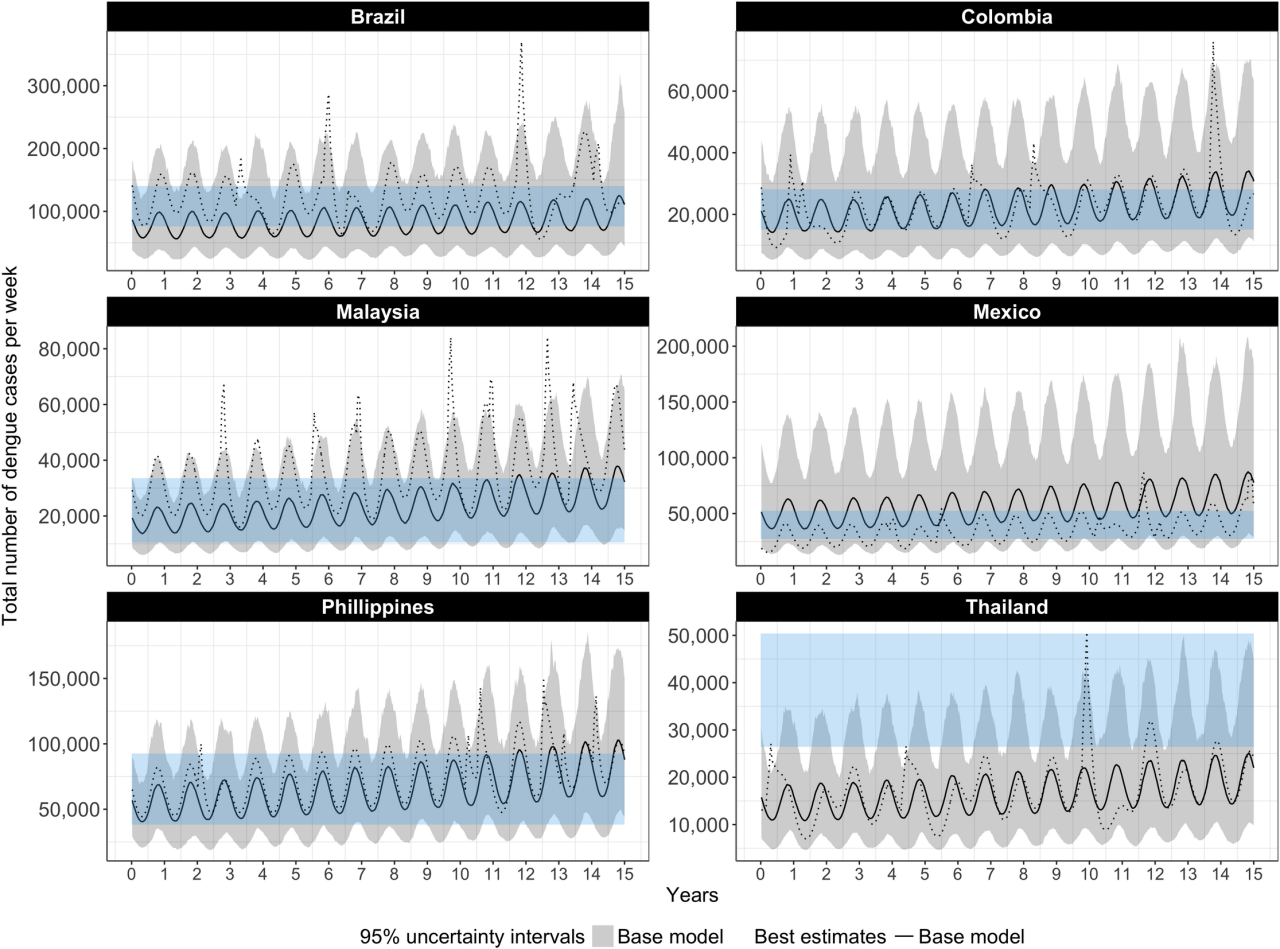
Weekly number of dengue cases with no vector control or immunization (case management only), our base model compared to published estimates of apparent cases, best estimates and 95% UIs. The black line and grey bands represent the best estimates and uncertainty intervals for the number of dengue cases as projected by our base model; the dotted black line represents the number of dengue cases in a single, randomly selected iteration of the model; the blue bands represent uncertainty intervals for apparent cases, as published by Bhatt et al. (2013)

The number of cases of symptomatic dengue in the baseline model (medical case management alone) is depicted in Fig 1, for each of the six countries. The waves represent seasonal variation. Outbreaks are not visible in the best estimate or uncertainty intervals obtained from the 1000 simulations but are visible in individual simulations, only one of which is depicted for illustration. We compare these baseline model results to recent published estimates to ensure that we are not overstating the potential effects of intervention in terms of cases or DALYs averted [22]. For most countries our estimates in year one are towards the lower bound of those published estimates, and for all countries the uncertainty intervals overlap. Our estimates trend slightly upward over time. Note that the published estimates take into account current (but variable) efforts to control dengue whereas the estimates presented in Fig 1 are for our baseline model (medical case management alone).

**Fig 2:**
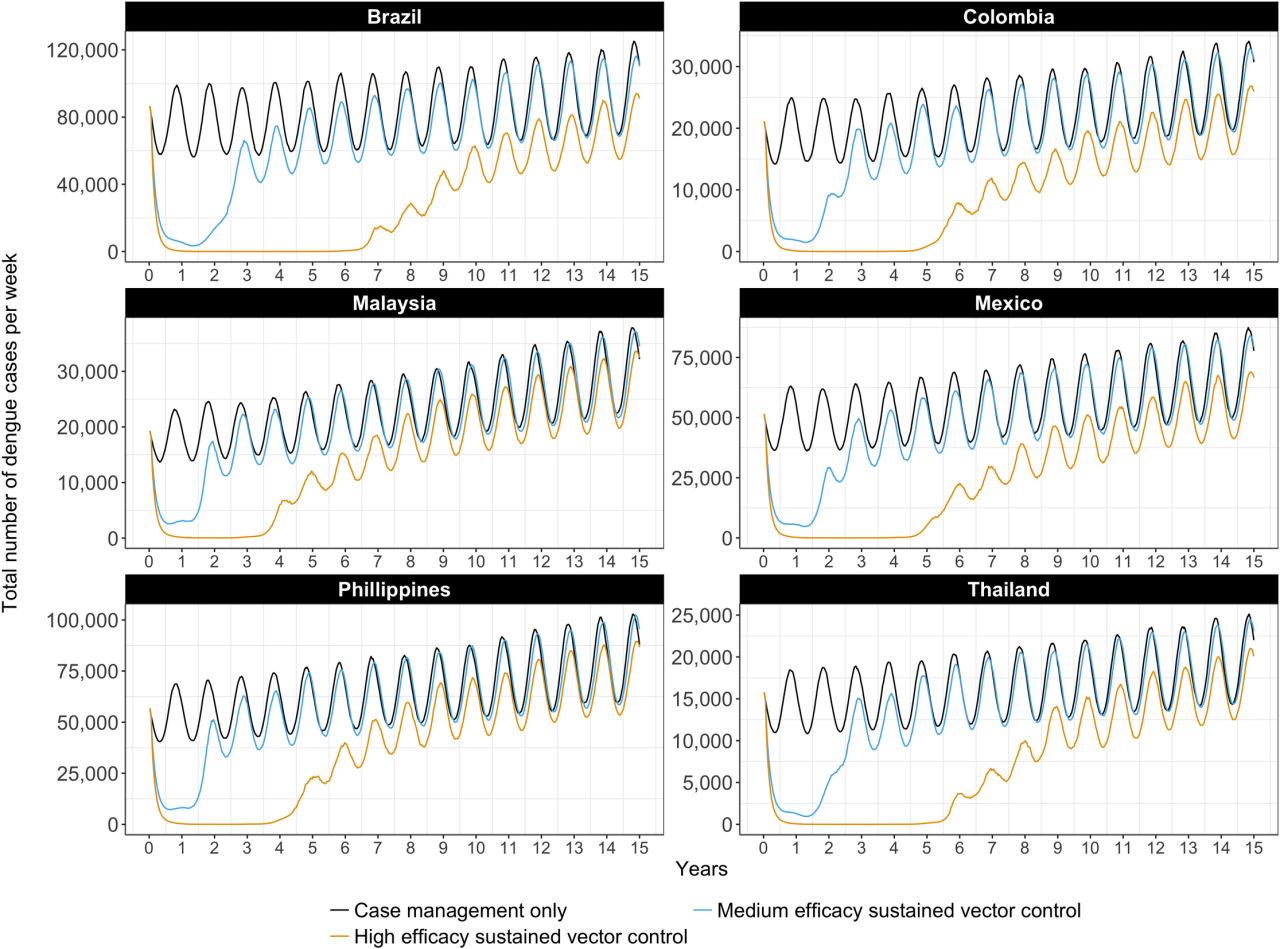
Weekly number of dengue cases with medium or high efficacy sustained vector control technologies but no immunization, best estimates.

The effect of sustained vector control on the total number of dengue cases (including severe cases) is depicted over time in Fig 2. Different sustained vector control technologies (medium and high efficacy) are considered, and compared to the baseline (medical case management only). Vector control technologies of low efficacy (<50% reduction in vector populations) have limited longer-term impact on transmission, and are therefore not depicted.

Initially, medium- and high-efficacy vector control technologies result in a significant decrease in the number of cases. The combination of vector control and acquired immunity push the basic reproduction number to below one. In time, the number of susceptible people waxes and herd immunity wanes. With high-efficacy vector control, the period of low transmission lasts from four to seven years, varying between countries. Differences between countries are explained by differences in the country-specific parameters, namely birth rates, death rates, and urban growth rates, which together determine the rate at which susceptible people are introduced into the model.

The number of cases of severe dengue follows a similar pattern, but with a somewhat longer-term effect of high efficacy vector control, as can be verified in Supporting Information S3 Fig.

**Fig 3:**
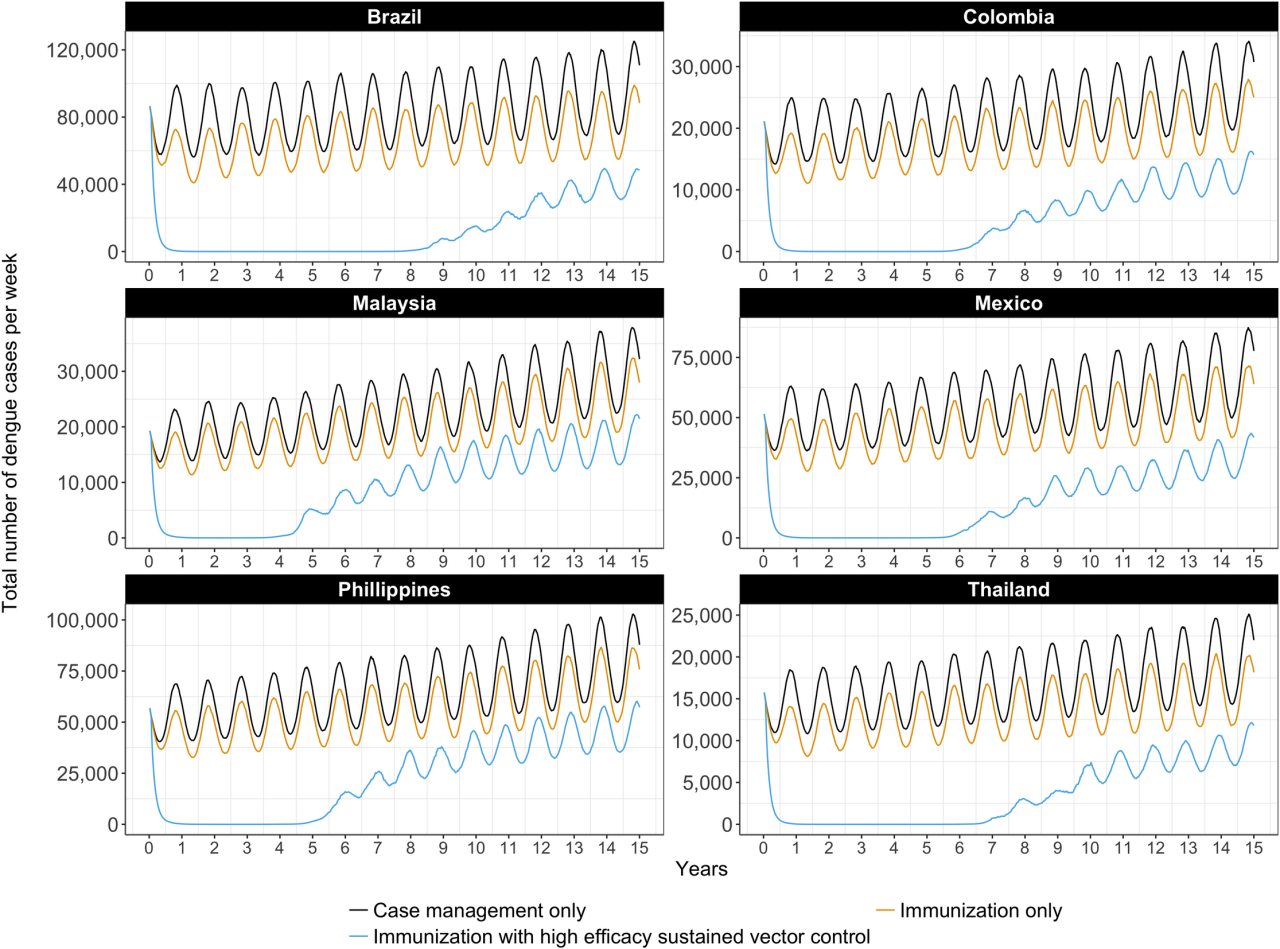
Weekly number of dengue cases with high efficacy sustained vector control and/or a highly targeted immunization strategy, best estimates.

The effect of highly targeted immunization using a medium-efficacy vaccine on the number of dengue cases is depicted over time in Fig 3, alone and in combination with sustained vector control (high efficacy). The total number of dengue cases is largely unaffected by immunization alone, because it is targeted at seropositives only and the number of severe dengue cases is small relative to the total. Only the combination of sustained vector control and immunization maintains the number of cases at very low levels, for between four and nine years depending on the country. Again, differences between countries can be explained by differences in the country-specific epidemiological parameters.

The number of cases of severe dengue, however, exhibits a different trend, as can be seen in Supporting Information S4 Fig. Here, because of our optimistic assumptions about how effectively an immunization strategy could target the seropositive population, we have a large and sustained effect of immunization, alone and in combination with sustained vector control (high efficacy). This figure should be interpreted as confirmation that our assumptions about the immunization strategy have been optimistic.

Table 4 summarizes the average annual number of DALYs in the period 2015–2030 under different intervention scenarios, compared to WHO Global Health Estimates for the year 2012. Our model suggests that medical case management alone would result in an average of 22.3–232.3 thousand DALYs per year (range of best estimates across the six countries). The uncertainty intervals on these estimates lie just above WHO estimates for the year 2012, with the exception of the Philippines (for which our uncertainty interval overlaps with the WHO estimate). Life expectancy is lower in the Philippines (69 years), than for any of the other countries (74–78 years). Note that WHO Global Health Estimates reflect variable levels of intervention across countries; they are also based on highest observed life expectancy globally.

**Table 4:** Average annual DALYs (in thousands) implied by the model (best and 95% uncertainty interval).

Country	Burden of disease^1^	Medical case management only			Outbreak response			Sustained vector control (medium efficacy technology)			Sustained vector control (high efficacy technology)		
	Best	Best	Low	High	Best	Low	High	Best	Low	High	Best	Low	High
BRA	103.0	232.3	99.4	539.7	232.2	98.9	537.2	167	18.6	427.5	89.6	1.4	341
COL	9.9	35.6	12.6	93	35.3	12.5	92.3	27.5	5.3	76.4	16.6	0.2	62.3
MEX	5.4	23.9	7.9	62.2	23.7	7.9	61	19.2	4.9	51.3	12	0.1	40.9
MYS	8.4	22.3	9.5	50.4	22.1	9.5	50.4	18.5	5	43.1	12.7	0.2	35
PHL	94.6	58.5	28.2	116.3	58.4	28.1	116.3	48.1	15.5	100	32.9	0.6	86.7
THA	9.5	26.6	9.5	67	26.4	9.4	66.9	20.2	4.2	52.7	11.9	0.2	41

^**1**^ WHO’s Global Health Estimates (2014) for the year 2012 [76].

Brazil (BRA), Colombia (COL), Mexico (MEX), Malaysia (MYS), Philippines (PHL) and Thailand (THA).

Outbreak response has a negligible impact on the average burden, given that outbreaks occur relatively infrequently on average and that the response is triggered only with a lag after the increase in the vector population. The introduction of sustained vector control (medium efficacy) reduces the burden to an average of 18.5–167.0 thousand DALYs per year. Sustained vector control (high efficacy) reduces the burden to an average of 12.0–89.6 thousand DALYs per year.

### Costs and affordability

Average annual costs over the 15-year period are reported in Table 5. At current prices of existing technologies, the total cost of sustained vector control (using a high efficacy technology) including outbreak response and medical case management (2013 $US 58.0–377.6 million, range of best estimates across the six countries) is comparable to what would have to be spent on outbreak response and medical case management (2013 $US 57.7–368.7 million). This result is driven by differences in the cost of treating the cases that would not be averted by outbreak response, given a minimum one week lag between the increase in vector populations and deployment of the outbreak response.

**Table 5:** Average annual cost and affordability, 2013 US$ millions.

**Country**	**Medical case management only**				**Outbreak response**				**Sustained vector control** **(high efficacy technology)**			
	Best	Low	High	% of GHE^1^	Best	Low	High	% of GHE^1^	Best	Low	High	% of GHE^1^
BRA	108	33.5	362.2	0.1	368.7	203.8	731.2	0.4	377.6	206.9	721.7	0.4
COL	74.3	22.6	234.6	0.4	124.1	49.4	293	0.6	105.5	37.8	253.7	0.5
MEX	175	50.2	591.9	0.5	326.6	121.3	808.2	0.9	276.3	104.7	698.4	0.7
MYS	43.6	16.3	104.5	0.6	86.9	42.4	156.7	1.3	84.5	38.1	151.7	1.2
PHL	66.2	27.3	158.1	1.8	148.1	75.9	255.2	4	149.5	70.1	271	4
THA	24.4	10.2	62.2	0.2	57.7	30.4	102.6	0.4	58	29.1	104.5	0.4

^**1**^ Best estimate divided by Government Health Expenditure (GHE).

Brazil (BRA), Colombia (COL), Mexico (MEX), Malaysia (MYS), Philippines (PHL) and Thailand (THA).

In Table 5, the best estimates of cost are expressed also as a percentage of government health expenditure (GHE) in 2013. Sustained vector control (high efficacy) including medical case management would cost 0.4–1.2% of GHE in five of the six countries. In the Philippines, where GHE in 2013 was much lower in per capita terms than for the other five countries, it would cost 4.0% of GHE.

### Cost-effectiveness

Average cost-effective ratios (ACERs) and incremental cost-effectiveness ratios (ICERs) are presented for each country in Tables 6 and 7, considering high- and medium-efficacy vector control technologies, respectively.

**Table 6:** Best estimates of costs, DALYs, Average Cost-Effectiveness Ratio (ACER) and Incremental Cost-Effectiveness Ratio (ICER), high efficacy vector control over 15 years.

Country (GDP pc 2013 US$)	Intervention	Costs (2013 US$ millions)	DALYs (thousands)	ACER (2013 US$)	ICER (2013 US$)
BRA (10958)	No intervention	0	6158.8	NA	NA
	Medium efficacy vaccine	1208.6	1967.7	288	288
	Case management	1620.5	3484.4	606	Dominated
	Medium efficacy vaccine and high efficacy vector control	5328.8	369.5	920	2578
	Just outbreak control	5530.3	3483	2067	Dominated
	High efficacy vector control	5664.2	1343.4	1176	Dominated
COL (7831)	No intervention	0	1437.9	NA	NA
	Medium efficacy vaccine	918.1	336.4	834	834
	Case management	1115.2	534	1234	Dominated
	Medium efficacy vaccine and high efficacy vector control	1349.3	80.6	994	1685
	High efficacy vector control	1582.2	249.3	1331	Dominated
	Just outbreak control	1861.1	529.8	2049	Dominated
MEX (11224)	No intervention	0	3766	NA	NA
	Medium efficacy vaccine	2152.6	247.7	612	612
	Case management	2625.7	358.9	771	Dominated
	Medium efficacy vaccine and high efficacy vector control	3644	87.1	991	9288
	High efficacy vector control	4144.3	179.9	1156	Dominated
	Just outbreak control	4899	355.7	1437	Dominated
MYS (10429)	No intervention	0	1422.9	NA	NA
	Medium efficacy vaccine	550	236.5	464	464
	Case management	654.3	334.1	601	Dominated
	Medium efficacy vaccine and high efficacy vector control	1137.5	72.9	843	3591
	High efficacy vector control	1267.2	190.6	1028	Dominated
	Just outbreak control	1302.8	331.8	1194	Dominated
PHL (2792)	No intervention	0	3794.8	NA	NA
	Medium efficacy vaccine	819.1	608.1	257	257
	Case management	992.6	878.2	340	Dominated
	Medium efficacy vaccine and high efficacy vector control	2024.5	186.3	561	2857
	Just outbreak control	2221.5	875.5	761	Dominated
	High efficacy vector control	2242.6	493.1	679	Dominated
THA (5879)	No intervention	0	1065.6	NA	NA
	Medium efficacy vaccine	289	245.4	352	352
	Case management	365.4	398.9	548	Dominated
	Medium efficacy vaccine and high efficacy vector control	795.6	53.4	786	2639
	Just outbreak control	866.1	396.5	1294	Dominated
	High efficacy vector control	869.4	179.1	981	Dominated

Brazil (BRA), Colombia (COL), Mexico (MEX), Malaysia (MYS), Philippines (PHL) and Thailand (THA).

**Table 7:** Best estimates of costs, DALYs, Average Cost-Effectiveness Ratio (ACER) and Incremental Cost-Effectiveness Ratio (ICER), medium efficacy vector control over 15 years.

Country (GDP pc 2013 US$)	Intervention	Costs (2013 US$ millions)	DALYs (thousands)	ACER (2013 US$)	ICER (2013 US$)
BRA (10958)	No intervention	0	6158.8	NA	NA
	Medium efficacy vaccine	1208.6	1967.7	288	288
	Case management	1620.5	3484.4	606	Dominated
	Just outbreak control	5530.3	3483	2067	Dominated
	Medium efficacy vaccine and medium efficacy vector control	5731.7	917.9	1094	4309
	Medium efficacy vector control	6189.6	2504.8	1694	Dominated
COL (7831)	No intervention	0	1437.9	NA	NA
	Medium efficacy vaccine	918.1	336.4	834	834
	Case management	1115.2	534	1234	Dominated
	Medium efficacy vaccine and medium efficacy vector control	1667.7	175.7	1321	4664
	Just outbreak control	1861.1	529.8	2049	Dominated
	Medium efficacy vector control	1954.4	412.9	1907	Dominated
MEX (11224)	No intervention	0	3766	NA	NA
	Medium efficacy vaccine	2152.6	247.7	612	612
	Case management	2625.7	358.9	771	Dominated
	Medium efficacy vaccine and medium efficacy vector control	4308.9	151	1192	22309
	Just outbreak control	4899	355.7	1437	Dominated
	Medium efficacy vector control	4995.2	287.4	1436	Dominated
MYS (10429)	No intervention	0	1422.9	NA	NA
	Medium efficacy vaccine	550	236.5	464	464
	Case management	654.3	334.1	601	Dominated
	Medium efficacy vaccine and medium efficacy vector control	1274.7	139.8	994	7499
	Just outbreak control	1302.8	331.8	1194	Dominated
	Medium efficacy vector control	1426.1	277.7	1245	Dominated
PHL (2792)	No intervention	0	3794.8	NA	NA
	Medium efficacy vaccine	819.1	608.1	257	257
	Case management	992.6	878.2	340	Dominated
	Just outbreak control	2221.5	875.5	761	Dominated
	Medium efficacy vaccine and medium efficacy vector control	2241	357.5	652	5673
	Medium efficacy vector control	2484.1	721.7	808	Dominated
THA (5879)	No intervention	0	1065.6	NA	NA
	Medium efficacy vaccine	289	245.4	352	352
	Case management	365.4	398.9	548	Dominated
	Just outbreak control	866.1	396.5	1294	Dominated
	Medium efficacy vaccine and medium efficacy vector control	886.5	124.6	942	4946
	Medium efficacy vector control	982.9	303.6	1290	Dominated

Brazil (BRA), Colombia (COL), Mexico (MEX), Malaysia (MYS), Philippines (PHL) and Thailand (THA).

If sustained vector control is effective in reducing mosquito populations by 70–90% (high-efficacy, Table 6), the average cost per DALY averted would be US$ 679–1331 (range of best estimates across the six countries) relative to the null scenario. The combination of high-efficacy sustained vector control and a highly targeted and low-cost immunization strategy using a medium-efficacy vaccine dominates all other interventions except immunization alone. However, immunization alone averts far fewer DALYs. In five of the six countries, the combination of vector control and immunization is very cost-effective, with an ICER well below one times GDP per capita. In the Philippines, it is cost-effective at a threshold just above one times GDP per capita. Recall that the Philippines is the country with the lowest life expectancy among the six; it is also the country with lowest GDP per capita, and therefore the country with the (presumed) lowest willingness-to-pay (WTP).

If vector control is effective in reducing mosquito populations by only 50–70% (medium-efficacy, Table 7), the average cost per DALY averted would be US$ 808–1907 (range of best estimates across the six countries) relative to the null scenario. Again, the combination of medium-efficacy sustained vector control and a highly targeted and low-cost immunization strategy using a medium-efficacy vaccine dominates all other interventions except immunization alone. However, again, immunization alone averts far fewer DALYs. In four of the six countries, the combination of medium-efficacy vector control and immunization is very cost-effective (the ICER is below one times GDP per capita). In Mexico and the Philippines it is cost-effective at thresholds between two and three times GDP per capita. Mexico is the country with the lowest reported death rate among severe dengue cases receiving medical management.

**Fig 4:**
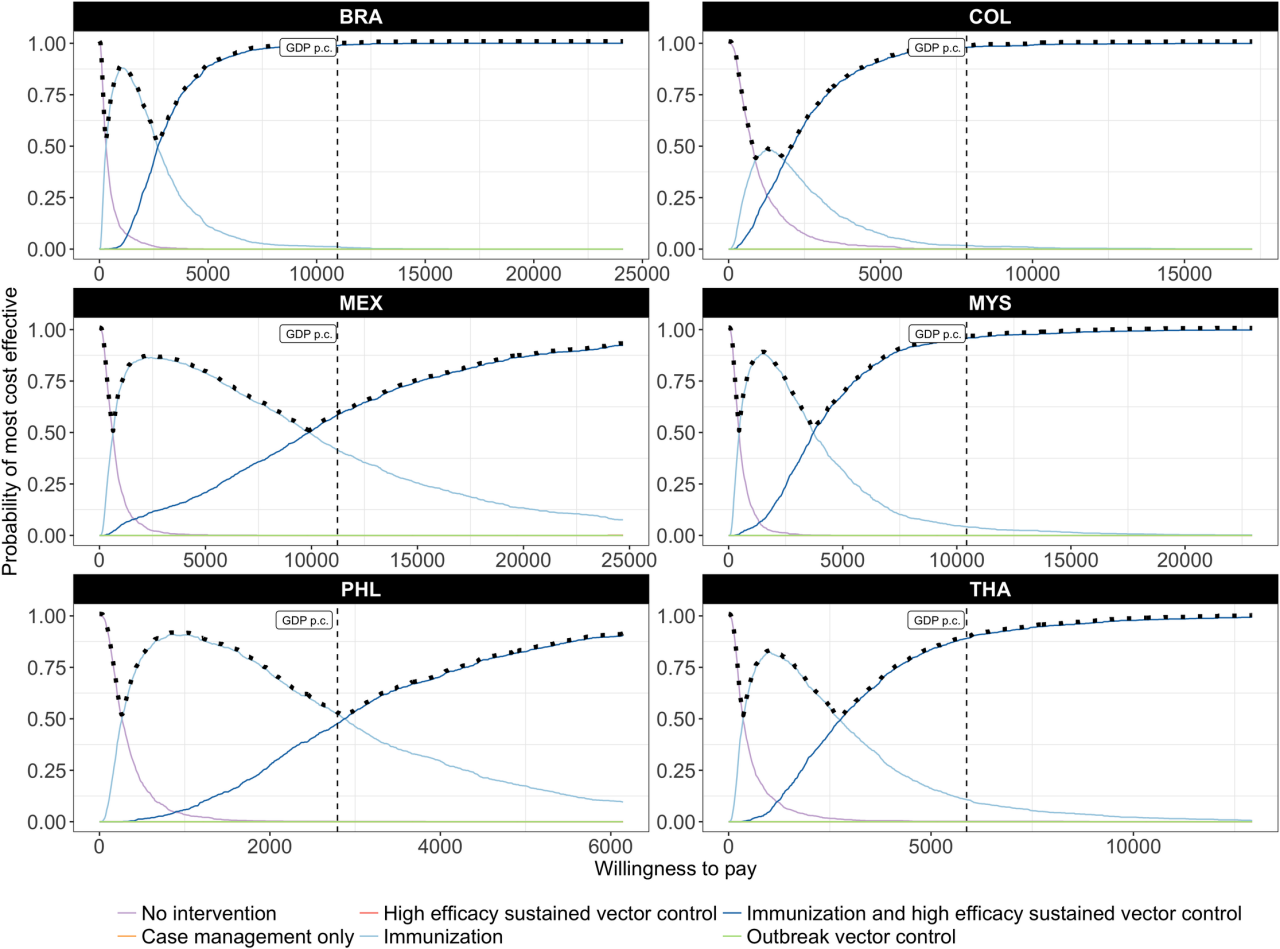
Probability of being most cost-effective at any given threshold, considering high-efficacy sustained vector control and a highly targeted, low-cost immunization strategy. The solid lines are cost-effectiveness acceptability curves (CEACs) representing the probability that an intervention is cost-effective at a given threshold; the (vertical) dashed line indicates the probability that an intervention is cost-effective at a threshold equal to GDP per capita; the dotted line represents the cost-effectiveness acceptability frontier (CEAF) which represents the probability that the most cost-effective option is cost-effective at a given threshold.

**Fig 5:**
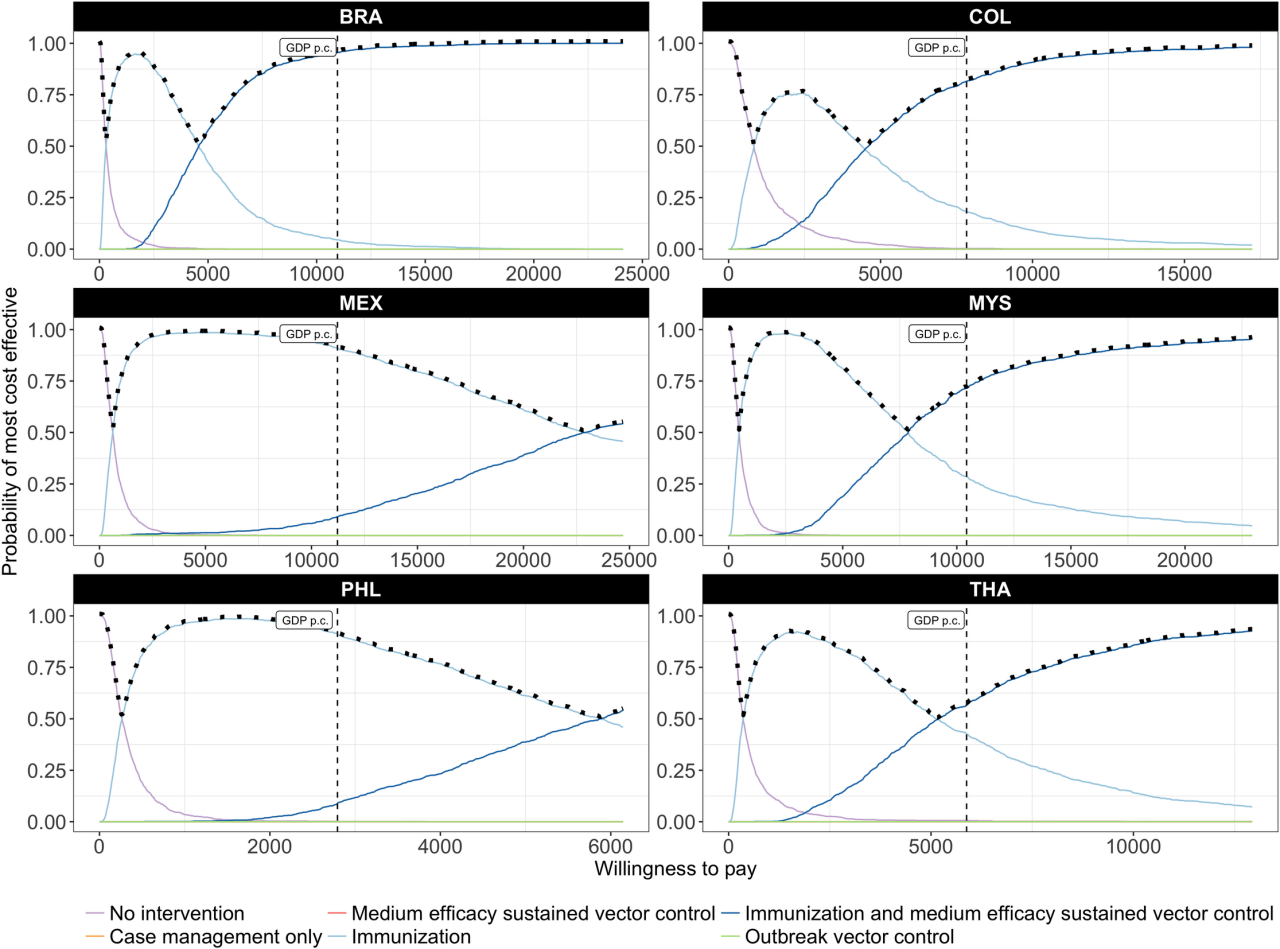
Probability of being most cost-effective at any given threshold, considering medium-efficacy sustained vector control and a highly targeted, low-cost immunization strategy. The solid lines are cost-effectiveness acceptability curves (CEACs) representing the probability that an intervention is cost-effective at a given threshold; the (vertical) dashed line indicates the probability that an intervention is cost-effective at a threshold equal to GDP per capita; the dotted line represents the cost-effectiveness acceptability frontier (CEAF) which represents the probability that the most cost-effective option is cost-effective at a given threshold.

Uncertainty around cost-effectiveness is reflected in the cost-effectiveness acceptability curves of Figs 4 and 5. The combination of high-efficacy sustained vector control and a highly targeted and low-cost immunization strategy using a medium efficacy vaccine (Fig 4) has the highest probability of being most cost-effective at WTP thresholds as low as one quarter of GDP per capita (per DALY averted). At a WTP threshold of one times GDP per capita, the probability that this is the most cost-effective strategy exceeds 85% in four of the six countries.

## Discussion

We estimated the cost and cost-effectiveness of sustained vector control in six high-burden countries. Our model suggests that, at current prices, the cost of sustained vector control and medical case management is comparable to what would otherwise have to be spent on outbreak response and medical case management. In December 2015, in its decision to approve Dengvaxia, Mexico reported that it was spending about 2.5% of its health budget on medical treatment alone [77]. Many countries have abandoned or lack effective surveillance systems to respond rapidly enough to outbreaks to make much of a dent in the number of cases requiring medical management [78].

Our results on cost are nonetheless conservative relative to an earlier review and qualitative synthesis of the evidence from single settings, which found that the cost of outbreak response exceeds that of sustained vector control [79]. We considered only direct medical costs and control programme costs–we did not consider direct non-medical costs faced by patients during their care (e.g. food, transportation) nor any productivity losses (time spent away from work) of the patients or their caretakers.

Our estimates of the cost per DALY averted by sustained vector control (related to doing nothing) are lower than that of the DCP2, but higher than those from studies of single settings. In those studies, cost-effectiveness ratios ranged from 2005 US$ 227 (2013 US$ 344) per DALY averted by larval control in Cambodia to 2009 US$ 615–1267 (2013 US$ 802–1652) per DALY averted by adult mosquito control in Brazil [9][10]. Taking a broader societal perspective including productivity losses, the Cambodia programme cost only 2005 US$ 37 (2013 US$ 56) per DALY averted. Again, we did not consider these productivity losses–their measurement remains controversial, although few would argue that they are zero.

We have not (nor to our knowledge has any earlier study) considered the additional benefits of vector control targeted at dengue for the prevention of other diseases, such as Chikungunya, yellow fever and Zika fever, transmitted by the same vectors and (for all but yellow fever) lacking effective vaccines and specific treatment. These additional benefits of sustained vector control would increase its cost-effectiveness. Even with conservative assumptions, our results suggest that sustained vector control can be highly cost-effective.

Our model suggests that the introduction of highly targeted and low-cost immunization strategy using a medium-efficacy vaccine does not alter the conclusion that sustained vector control can be highly cost-effective. On the contrary, vector control may complement a medium-efficacy vaccine, or a vaccine that is highly effective but against only secondary infections or only one of the four dengue serotype, or whose production is highly constrained. In these instances, sustained vector control may compensate to some extent for lower levels of coverage by immunization.

There are as yet no costing studies for dengue vaccine delivery, nor even a known price for the vaccine itself [77][80][81]. Indeed, there are still many unknowns about immunization for dengue. Overall, our assumptions for immunization can be considered conservative from the perspective of the cost-effectiveness of sustained vector control. That is, we have been optimistic in our assumptions about immunization with regard to both effects and costs, in order to have the most conservative estimate of the cost-effectiveness of vector control in the presence of immunization. We have been particularly optimistic in assuming that the targeting strategies for immunization are perfectly effective in avoiding seronegative people. This analysis should not, therefore, be used to draw conclusions about the cost-effectiveness of the current vaccine. When new information or new vaccines become available, these model parameters should be adapted.

We accounted for many of the known sources of uncertainty within a probabilistic framework. In spite of considerable uncertainty, sustained vector control emerged with a high probability of cost-effectiveness. Some sources of uncertainty, however, could not be accounted for and remain as more serious limitations to our study.

First, there is remaining uncertainty around particular parameter values. The serotype immunity variable (serotype composition) is assumed to be constant over time. The probability that dengue fever following a second infection develops into severe dengue is based on clinical paediatric data from four hospitals [39]. While the probability of death among severe dengue cases receiving medical management was based on country-reported data, the considerable disparity across countries raises questions. The availability of better data for these parameters would improve the precision of our model.

Second, there is remaining uncertainty about the structure of the model itself, namely with respect to outbreaks, which are modelled as increases in the vector population. Vector indices have been correlated to increases in dengue cases during outbreaks, but the strength of evidence is limited by a lack of well-controlled studies [82][83]. Other studies have considered spatial, meteorological, epidemiological, and entomological factors, and dengue serotype [84]. Although this study could benefit from a more sophisticated outbreak model, uncertainty remains in the choice of variables and availability of data.

Another limitation of the model structure is that we have not modelled heterogeneity in transmission. The probability of contracting dengue assumes an even distribution of vectors to humans throughout the susceptible population. However, a study from Armenia and Colombia found that 95% of *Ae aegytpi* pupae were concentrated in only 5% of houses [85]. More detailed risk mapping could improve our analysis and lead to better targeting and enhanced cost-effectiveness of vector control.

Third, we have made no assumptions about the relative costs of the different vector control technologies–in fact, we have assumed the same unit cost for both medium- and high-efficacy technologies. Obviously, this analysis does not help us to choose amongst available vector control technologies. Further research is needed to understand the drivers of cost and effect across current and future vector control technologies. Indeed, it is unclear whether reductions in excess of 70–90% can be sustained using existing technologies; more research on the long-term impact of both existing and upcoming technologies is needed.

Nonetheless, the result on the cost-effectiveness of high-efficacy vector control is fairly robust to higher unit costs. Comparing ICERs to WTP, we find that in most settings, the cost of sustained vector control using high-efficacy technologies could be considerably higher than that assumed in our model (about US$ 0.05 per capita per month) before it would no longer be considered cost-effective. At a WTP threshold of three times GDP per capita, the cost could be as much as 2.9–13.9 times higher without affecting our conclusions for any of the settings considered. At a WTP threshold of one times GDP per capita, the cost could be as much as 2.2–4.6 times higher without affecting our conclusions for Brazil, Colombia, Malaysia, and Thailand.

Fourth, our model does not reflect uncertainty around the impact of resistance to insecticides in a scenario of sustained vector control. The data on this are limited, as a result of poor routine resistance monitoring [86]. New vector control technologies, such as genetic modification or symbiont infection of vectors, once developed, may help in mitigating the risk of resistance [87]. Our results are favourable to sustained vector control in general (not to any one technology in particular) and should not discourage the development of new technologies with demonstrated efficacy and safety. Indeed, our results apply to any technology that reduces the number of vectors able to transmit disease, rather than the number of vectors per se.

Finally, our results may not be generalizable to other settings, for which we do not have good epidemiological or cost data. Low-income countries, especially those in Africa, are arguably more vulnerable and less prepared for the effects of unplanned urbanization and climate change, including the spread of vector-borne diseases [88]. These same countries have the poorest quality data on dengue, starting with the frequent misclassification of dengue as malaria. A regional study of the cost and cost-effectiveness of sustained vector control in low-income countries of Africa is needed.

This paper focussed on the cost-effectiveness of sustained vector control in six middle-income endemic countries representing 15% of the estimated global burden of dengue. We have shown that sustained vector control will be cost-effective in most of these countries if it succeeds in reducing mosquito populations by more than 50%. Importantly, we show that the introduction of a highly targeted and low-cost immunization strategy using a medium-efficacy vaccine does not weaken the investment case for sustained vector control. Middle-income endemic countries should proceed with mapping the populations to be covered by sustained vector control.

## Supporting information

S1 Fig Markov model of dengue infection in human and vector populations. (PNG)

S2 Fig Relationship between cost per capita per month (2013 US$) and population covered by sustained vector control. (TIFF)

S3 Fig Weekly number of severe dengue cases with medium or high efficacy sustained vector control technologies but no immunization, best estimates. (TIF)

S4 Fig Weekly number of severe dengue cases with medium or high efficacy sustained vector control and/or immunization, best estimates. (TIF)

S1 Table Result from meta-regression of the cost per capita per month (2013 US$) of sustained vector control. (DOCX)
