# Supplementary figures and images for: An economic evaluation of vector control in the age of a dengue vaccine
Source: PLoS Negl Trop Dis. 2017 Aug 14;11(8):e0005785. doi: 10.1371/journal.pntd.0005785 (PMC5573582; doi:10.1371/journal.pntd.0005785)

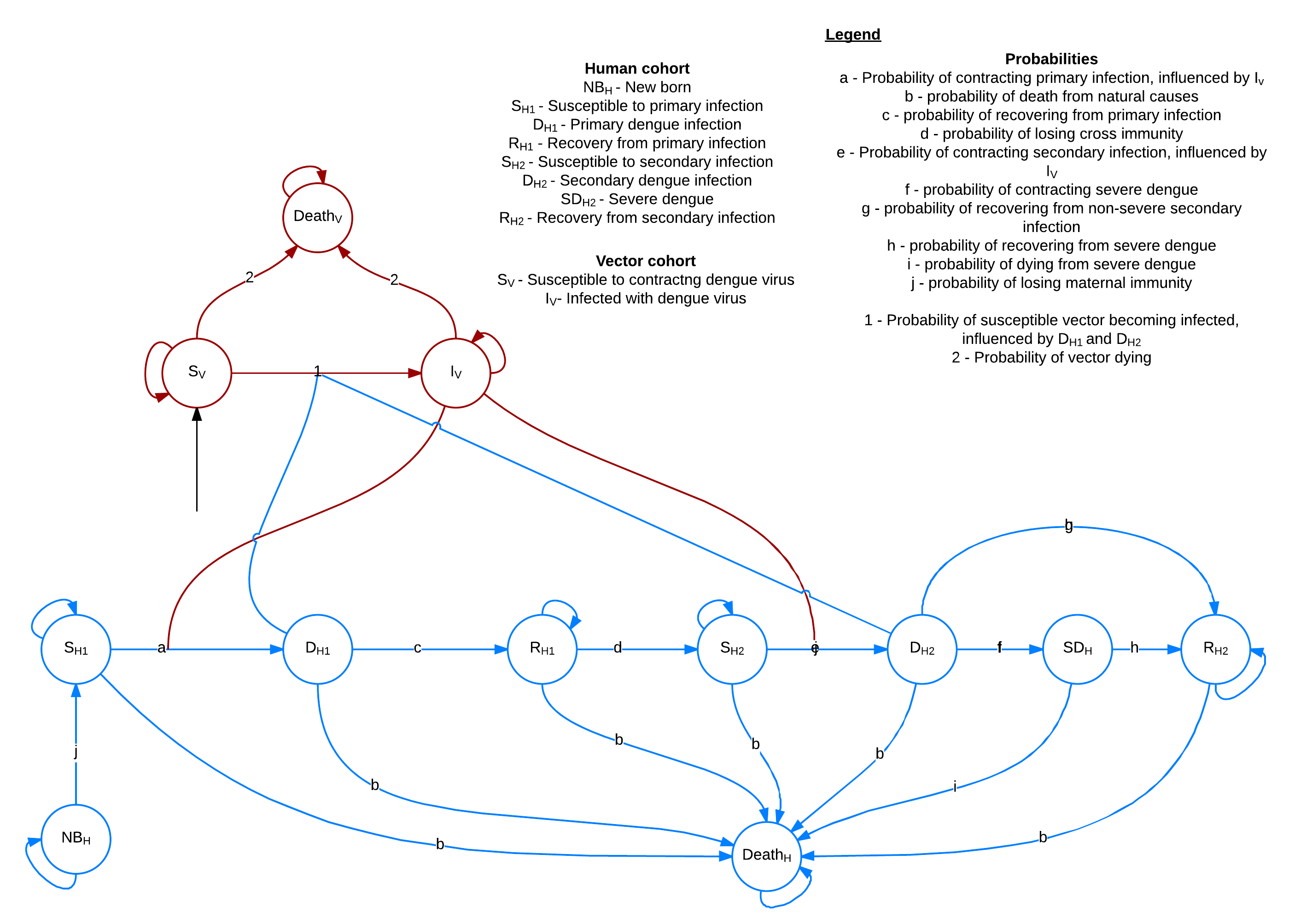

Supplement: S1 Fig — (PNG) [file pntd.0005785.s001.png]

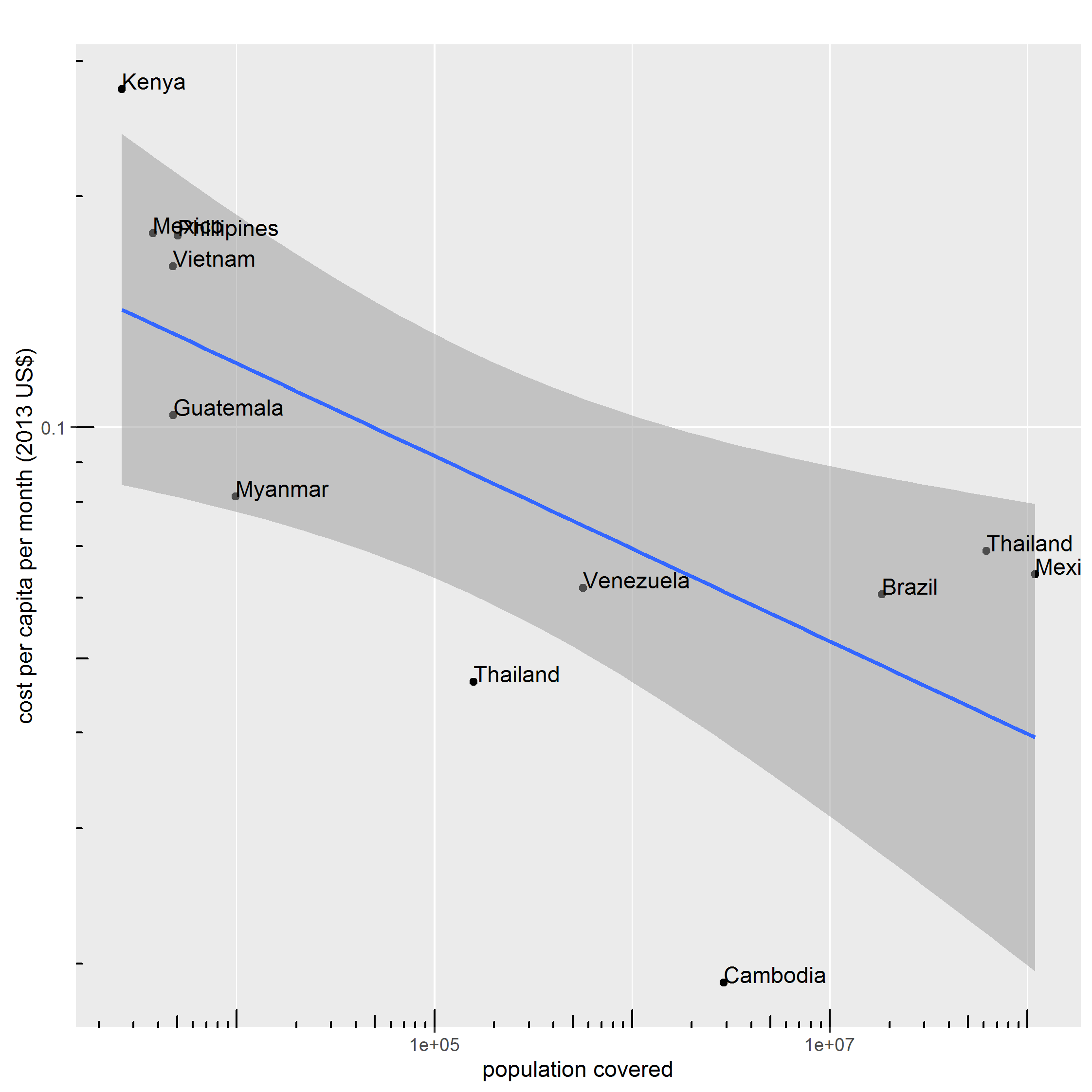

Supplement: S2 Fig — (TIFF) [file pntd.0005785.s002.tiff]

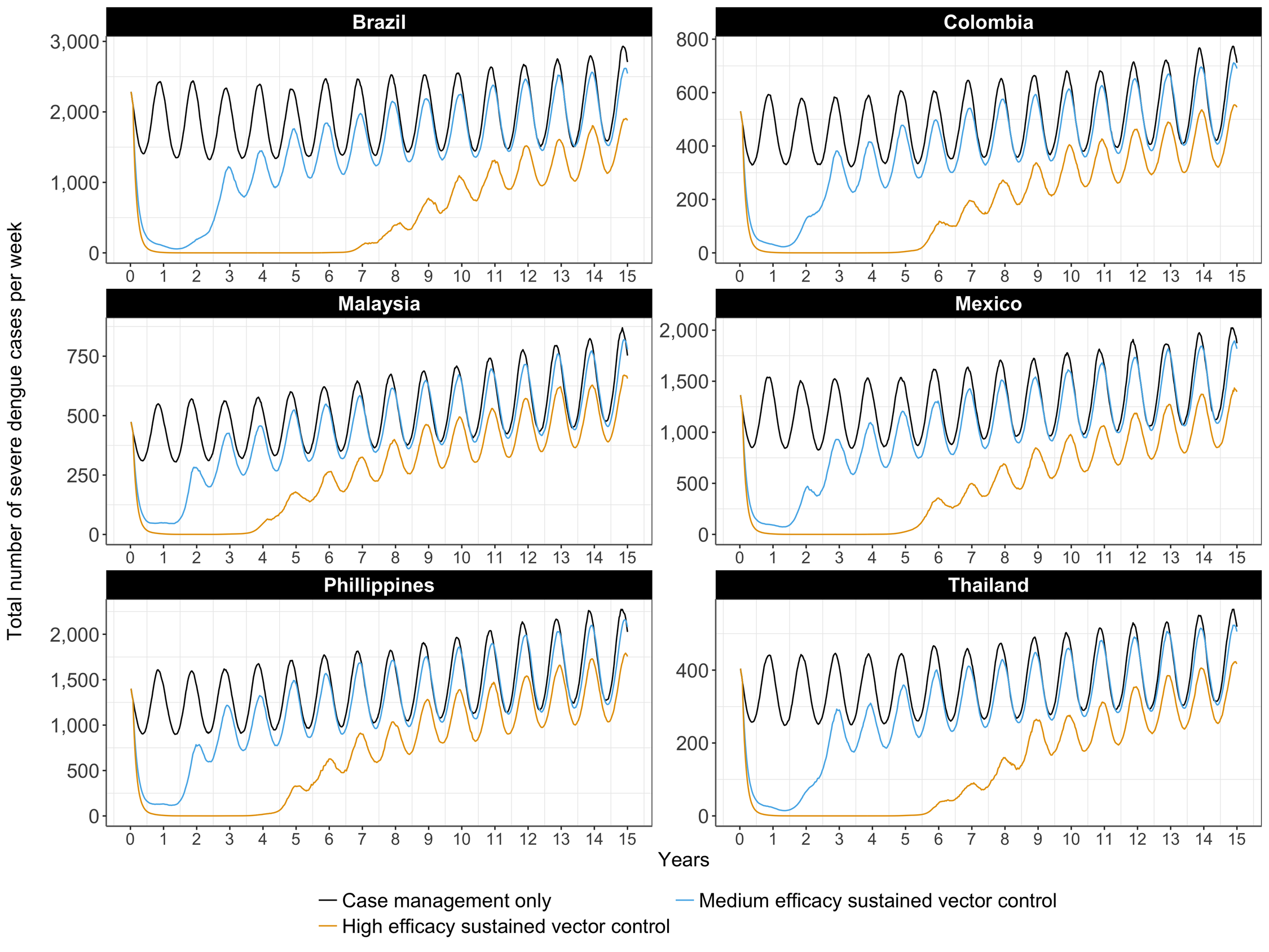

Supplement: S3 Fig — (TIF) [file pntd.0005785.s003.tif]

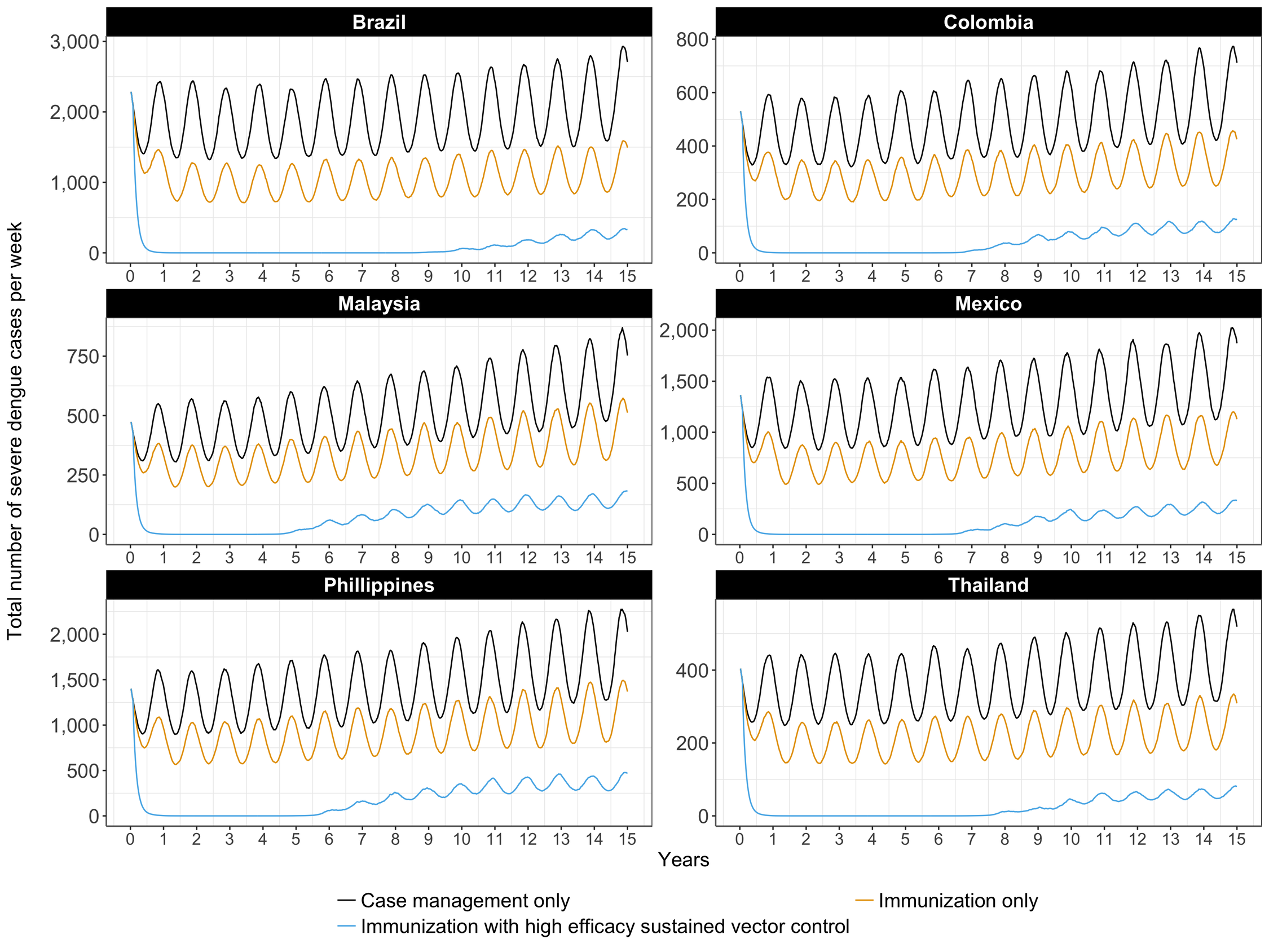

Supplement: S4 Fig — (TIF) [file pntd.0005785.s004.tif]
