# Supplementary material for: An economic evaluation of vector control in the age of a dengue vaccine
Source: PLoS Negl Trop Dis. 2017 Aug 14;11(8):e0005785. doi: 10.1371/journal.pntd.0005785 (PMC5573582; doi:10.1371/journal.pntd.0005785)
Supplement: S1 Table — (DOCX) [file pntd.0005785.s005.docx]

|  | |
| --- | --- |
|  | *Dependent variable:* |
|  |  |
|  | log(unit cost) |
|  | |
| log(population) | -0.159^**^ (0.050) |
| log(GDP per capita) | 0.145 (0.153) |
| Constant | -1.729 (1.166) |
|  | |
| Observations | 11 |
| R^2^ | 0.560 |
| Adjusted R^2^ | 0.450 |
| Residual Std. Error | 0.565 (df = 8) |
| F Statistic | 5.094^**^ (df = 2; 8) |
|  | |
| *Note:* | ^*^p<0.1; ^**^p<0.05; ^***^p<0.01 |
